# Dynamic pruning group equivariant network for motor imagery EEG recognition

**DOI:** 10.3389/fbioe.2023.917328

**Published:** 2023-05-26

**Authors:** Xianlun Tang, Wei Zhang, Huiming Wang, Tianzhu Wang, Cong Tan, Mi Zou, Zihui Xu

**Affiliations:** ^1^ Department of Computer Science and Technology, Chongqing University of Posts and Telecommunications, Chongqing, China; ^2^ Xinqiao Hospital, Army Medical University, Chongqing, China

**Keywords:** motor imagery, group convolution network, prune, short-time Fourier transform, deep learning, BCI

## Abstract

**Introduction:** The decoding of the motor imaging electroencephalogram (MI-EEG) is the most critical part of the brain-computer interface (BCI) system. However, the inherent complexity of EEG signals makes it challenging to analyze and model them.

**Methods:** In order to effectively extract and classify the features of EEG signals, a classification algorithm of motor imagery EEG signals based on dynamic pruning equal-variant group convolutional network is proposed. Group convolutional networks can learn powerful representations based on symmetric patterns, but they lack clear methods to learn meaningful relationships between them. The dynamic pruning equivariant group convolution proposed in this paper is used to enhance meaningful symmetric combinations and suppress unreasonable and misleading symmetric combinations. At the same time, a new dynamic pruning method is proposed to dynamically evaluate the importance of parameters, which can restore the pruned connections.

**Results and Discussion:** The experimental results show that the pruning group equivariant convolution network is superior to the traditional benchmark method in the benchmark motor imagery EEG data set. This research can also be transferred to other research areas.

## 1 Introduction

The brain-computer interface (BCI) allows the human brain to directly interact with computers or other external devices using electroencephalogram (EEG) signals. The research of the human BCI mainly includes three types: the invasive BCI, partial BCI, and non-invasive BCI. Although there are many BCI systems and technologies, the non-invasive BCI (EEG) has been widely studied because of its low cost, simplicity, and good time resolution. Motor imaging (MI) is one of the most widely studied BCI applications based on EEG, which can help the disabled and elderly to complete specific tasks through imagination without using limbs (Lotte et al., 2007; Pfurtscheller et al., 2006).

**FIGURE 1:**
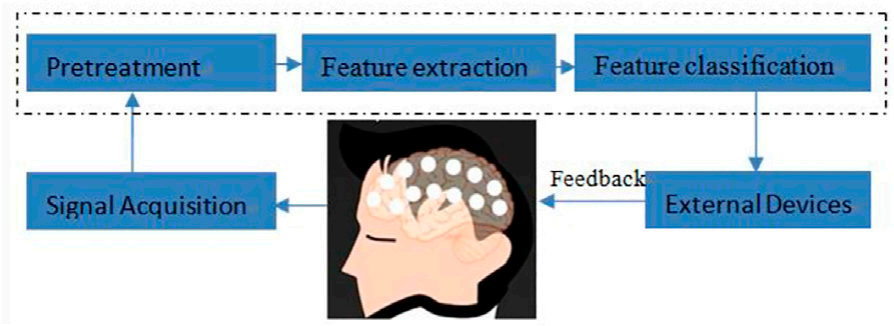
BCI system.

The design of a typical EEG-based brain-computer interface system for motor imagination is shown in Figure 1. Generally speaking, the BCI system of motor imagination is mainly composed of five parts: signal data acquisition, data preprocessing, feature extraction, feature classification and the equipment control interface (Subasi, 2007). The data acquisition stage includes MI-EEG signal acquisition and analog-to-digital conversion through an EEG cap. Because the amplitude of the EEG signal is very weak, it is easily affected by EMG, eye electricity, and AC power frequency interference. In the data preprocessing stage, time-domain filtering and spatial filtering are carried out to improve its signal-to-noise ratio. In the feature extraction stage, the features for specific tasks are extracted from EEG data for classification. In the feature classification stage, a machine learning algorithm is used to decode its meaning from EEG features. Finally, in the equipment control interface stage, instructions are sent to peripherals, such as computers, wheelchairs, and robots, according to the meaning of the EEG signals (Bhattacharyya et al., 2019; Bigirimana et al., 2020; Li et al., 2019; Orset et al., 2021; Shi et al., 2020; Song and Kim, 2019).

The theoretical basis of MI signal generation is event-related desynchronization and event-related synchronization. When people imagine a limb movement in their brain, the corresponding area of the sensorimotor cortex will be in an active state, and the alpha and beta waves in the EEG signals generated by this area will be attenuated in amplitude, which is called event-related desynchronization. On the contrary, if the brain does not carry out the motor imagery task, the amplitude of the alpha and beta spectrum concussion of the EEG will be significantly enhanced, which is called event-related synchronization. At present, there are many research studies on the BCI of motor imagination, such as left hand movement, right hand movement, leg movement, and tongue movement.

For a long time, many research studies on the EEG interface have been devoted to feature extraction and classification because they have a crucial impact on the performance of the BCI system. The common space pattern (CSP) algorithm is a classical algorithm used to extract the characteristics of original EEG signals. The CSP algorithm and several improved algorithms have been developed and applied to the BCI of motor imagination (Wang et al., 2006) (Aghaei et al., 2013). For example, Aghaei et al. designed a separable common spatial spectral pattern algorithm for the BCI of motion imagination, which has certain advantages in computational complexity. In addition, in the EEG feature extraction stage, many time–frequency signal processing methods have also achieved good results, such as short-time Fourier transform (STFT), empirical mode decomposition (EMD), and continuous wavelet transform (CWT) (Lee and Choi, 2018; Park et al., 2013; Tabar and Halici, 2017). Tabar and Halici used STFT to integrate the time, frequency, and position information extracted from the original EEG signal and convert it into an image. Lee and Choi used continuous wavelet transform to transform EEG signals into a time–frequency spectrum. Sometimes, in order to improve the computational efficiency, a feature selection process is added to the extracted features to remove redundant information. In the feature classification stage, the commonly used algorithms are linear discriminant analysis (LDA), support vector regression (SVR), and artificial neural network (Naseer and Hong, 2013; Siuly and Li, 2012; Subasi, 2005). Naseer and Hong applied the LDA classifier to the motor imagination classification task based on two different features. Siuly and Li designed a least square support vector machine method to classify motion imagination signals. However, there is a common problem in these traditional algorithms, and they rely too much on the prior knowledge of EEG signal processing.

With the deepening of the application of the deep learning method in the field of EEG signal processing, the end-to-end learning method combining EEG feature extraction and classification shows obvious advantages. In recent years, many EEG classification methods based on the deep learning model show superior performance to traditional methods (Ek and Bma, 2021; Idowu et al., 2021; Sun et al., 2020; Sun et al., 2022a; Yang et al., 2015). As the first deep learning model introduced into EEG signal processing, the CNN integrates EEG feature extraction and classification and has achieved good final classification results. At the same time, graph convolutional network and transfer learning technology have also been introduced into brain computer interface research, and some new progress has also been made (Zhang et al., 2021; Sun et al., 2022b; Sun et al., 2023).

Although CNNs have achieved significant performance improvement on some benchmark problems, their training efficiency and generalization ability still need to be improved. One concept developed for this purpose is equivariant, which again draws inspiration from humans. Humans can recognize familiar objects, although they differ in location, size, angle of view, lighting conditions, and background. In addition, we can not only identify them accurately but also describe the types and parameters of relevant changes in detail (Schmidt et al., 2016). Equivariant is closely related to the concept of symmetry. Since these changes will not change the essence of the underlying objects, they should be treated and learned as a single concept. Recently, several methods have adopted these ideas to maintain symmetry, including translation (LeCun et al., 1989), scaling (Sosnovik et al., 2020), and general symmetry group (Bekkers, 2020; Romero and Hoogendoorn, 2020; Venkataraman et al., 2020).

Although a group convolution network (GCNN) can learn powerful representation based on symmetric patterns, it is an important model to improve the learning ability of small samples. However, the existing group convolution network model structure still has a large number of redundant weights, which is easy to overfit and difficult to deploy on the mobile platform with limited computing power. In this paper, a pruning group convolution network is proposed. By pruning the connections dynamically, the robustness of EEG recognition is significantly improved. Different from the previous greedy way to complete this task, we combine properly splicing the connections in the whole process to avoid incorrect pruning and make it a continuous dynamic update of network weights. The experimental results show that the method is effective. While improving the robustness of a small sample EEG recognition task, our method can effectively compress the number of parameters in the GCNN with a compression factor of 15 ×. This method is superior to the common GCNN to a great extent.

Specifically, we propose a robust group convolution based on dynamic pruning group convolution. In the process of dynamic pruning group convolution, pruning is used to emphasize meaningful symmetric combinations and suppress unreasonable, redundant, and possibly misleading combinations. In addition, we propose a new method of dynamic pruning and experimentally prove that our dynamic pruning equivariant group convolution network performs better than the traditional group equivariant network and other benchmark methods on BCI IV 2B and self-collected EEG datasets.

The contribution of this paper is as follows:

(1) A group convolution network based on time–frequency spatial EEG representation is proposed, which integrates the spatiotemporal spectral information of EEG signals into a unified network framework. The group convolution of the EEG feature extraction layer keeps equivariant under symmetric transformation, which constrains the network and improves the statistical efficiency, thus contributing to the generalization of network performance.
(2) We propose a general group theory framework about pruning, that is, dynamic pruning group convolution, which can adaptively capture the discriminative patterns in brain regions, frequency bands, and time domains, effectively compress the number of parameters in the GCNN, and dynamically evaluate the importance of parameters through dynamic pruning, so as to restore the pruned connections in time, reducing the number of parameters at the same time. It helps to find out the really important connections and improves the robustness of the model.
(3) Several experiments on two benchmark datasets show that our algorithm is always superior to state-of-the-art models.

The rest of this paper is arranged as follows. The second section briefly introduces the related work. The third section describes the process of EEG pruning group convolution network analysis and proposes a pruning group convolution (DPGEN) framework for EEG classification. In the fourth section, experiments are carried out on the open dataset BCI IV 2B and the self-collected dataset of the laboratory, and the experimental results are analyzed. Finally, the fifth section summarizes the paper and prospects the future work.

## 2 Related work

### 2.1 EEG-ConvNet

Recently, deep learning has attracted increasing attention in many types of machine learning problems in the medical field. The end-to-end training of deep neural networks (ConvNets) from original signals is a promising deep learning technology. These ConvNets utilize the hierarchical structure of many natural signals. A deep convolution neural network (EEG-ConvNet) for EEG recognition was proposed by Lawhern et al. (2016). The model eliminates the dependence on the channel layout by using spatial filtering in the first layer (Blankertz et al., 2008). Springenberg et al. (2014) focused on spatiotemporal convolution in spatial filter space to capture the spatiotemporal relationship of the EEG. At the same time, in order to reduce the total number of parameters, the model omits the fully connected layer. This model can be used together with a small EEG database and can improve the latest performance of multiple tasks and subjects in some cases, which challenges the concept that large datasets are required to obtain the best performance.

### 2.2 Group convolution network

Deep convolutional neural networks (CNNs) have been proved to be very powerful models, such as image, video, and audio sensory data. Convolution weight allocation and depth (and other factors) are important for good prediction performance. The convolution layer can be used effectively in an in-depth network. One important reason is that the convolution layer is translationally equivariant: moving the image and feeding it through several layers is the same as feature mapping (at least reaching the edge effect) obtained by feeding the original image through the same layer and then moving it. In other words, symmetry (translation) is maintained by each layer, which makes it possible to utilize symmetry not only at the first layer but also at higher levels of the network.

Cohen and Welling (2016) showed how to generalize convolutional networks to more general symmetric groups, including rotation and reflection transformations, and the concept of equivariant is the key to this generalization. For some selected groups, the model constructs a representation with a linear group space structure. This means that each vector in the representation space has an attitude associated with it, and this additional structure can more effectively model the data: convolution nuclear energy in the group convolution network detects the co-occurrence of features with priority-related attitude and can match such feature sets in each global attitude through an operation called group convolution.

In deep learning, general equivariant is more useful than invariance because it is generally impossible to determine whether features are in the correct spatial structure if they are invariant. In addition to improving statistical efficiency and promoting geometric reasoning, the equivariant of symmetric transformation constrains the network in a way conducive to generalization.

### 2.3 Neural network dynamic pruning strategy

As a brain-inspired model, the deep neural network (DNN) is widely used in image classification, natural language processing, speech recognition, and EEG recognition. Although DNN models usually need a large number of parameters to ensure their superior performance, there is significant redundancy in their parameters. Therefore, with appropriate strategies, these models can be compressed without significantly reducing the prediction accuracy. In the existing methods, network pruning has become a prominent method because of its amazing model compression ability under the condition of ensuring the prediction accuracy. For example, Han et al. (2015) proposed a lossless depth neural network compression method by eliminating redundant parameters and repeated iterative training.

However, due to the complex interconnection between hidden neurons, the weights of parameters may change significantly once pruning is performed. This leads to two main problems in some other classical methods. The first problem is that there is no way of recovering the possible network damage. Since there is no chance of repairing the trimmed connection, improper pruning may result in serious precision loss. Therefore, the compression ratio must be excessively suppressed to avoid this loss. Another problem is the low efficiency of learning. As described previously, in order to obtain the appropriate compression rate on AlexNet, it is necessary to alternate pruning and retraining several times, and each retraining process contains millions of iterations, which may be very time consuming. Mathieu et al. (2013) attempted to solve these problems and pursue the compression limit of the pruning method. It is recommended to cut off redundant connections through continuous network maintenance. This method involves two key operations: pruning and splicing. Obviously, pruning is performed to compress the network model, but overpruning or wrong pruning will lead to the loss of accuracy. In order to compensate for the unexpected loss, this method properly integrates the splicing operation into network pruning so that when the spliced connection is found to be important, the connection can be restored. These two operations are integrated to make the method dynamic by updating the parameter importance when necessary. Pruning and splicing naturally constitute a cycle, similar to the synthesis of excitatory and inhibitory neurotransmitters in the human nervous system.

## 3 Dynamic pruning group equivariant network

### 3.1 Equivariant group convolution network

Group convolution is the generalization of ordinary convolution on groups. We first introduce this *a priori* concept.

#### 3.1.1 Translation equivariant of ordinary convolution

Let $f,\psi :R^{d}\to R^{N_{c}}$ be a vector signal and a convolution kernel on R^d^ such that $f={\left\{f_{c}\right\}}_{c=1}^{N_{c}}$ and $\psi ={\left\{\psi_{c}\right\}}_{c=1}^{N_{c}}$ . Ordinary convolution (Blankertz et al., 2008) $\left({*}_{R^{d}}\right)$ is defined as

$$\left[f{*}_{R^{d}}\psi \right]\left(y\right)=\sum_{c=1}^{N_{c}}\int_{R^{d}}f_{c}\left(x\right)\psi_{c}\left(x-y\right)dx.$$ (1)

In order to study (and generalize) the properties of convolution, formula (1) is rewritten by using the translation operator $l_{y}$ :

$$\left[f{*}_{R^{d}}\psi \right]\left(y\right)=\sum_{c=1}^{N_{c}}\int_{R^{d}}f_{c}\left(x\right)l_{y}\psi_{c}\left(x\right)dx,$$ (2)

where $l_{y}\psi_{c}\left(x\right)=\psi_{c}\left(x-y\right)$ . It should be noted that the translation operator $l_{y}$ is indexed by the translation y. So, we actually consider a set of operators ${\left\{l_{y}\right\}}_{y\in R^{d}}$ , which index all possible translation sets y∈R^d^. A basic characteristic of convolution is that it can be exchanged with translation:

$$l_{y}\left[f{*}_{R^{d}}\psi \right]\left(x\right)=\left[l_{y}\left[f\right]{*}_{R^{d}}\psi \right]\left(x\right),x,y\in R^{d}.$$ (3)

In other words, the convolution of the y-translated signal $l_{y}\left[f\right]$ with a convolution kernel is equivalent to the convolution of original signal f with filter ψ and then y-translation. This property is called translational equivariant. Translation equivariant is considered to be the basis of good performance of the convolutional neural network in many application scenarios.

#### 3.1.2 Group convolution and group equivariant

Space convolution can be extended to general transformation, and a more general set ${\left\{l_{g}\right\}}_{g\in G},s.t\;{\left\{l_{y}\right\}}_{y\in R^{d}}\subseteq {\left\{l_{g}\right\}}_{g\in G}$ is used. However, in order to keep the equivariant, the transformation type allowed in ${\left\{l_{g}\right\}}_{g\in G}$ can be limited by the idea of group theory.

A group is a tuple (*G*,•), which is composed of a set G, g∈G, and a binary operation group product •:*G×G*→*G .* Let *f*,*ψ*:*G→* ***R*** ^*Nc*^ be the input signal and group convolution kernels on *G*, respectively, and group convolution (*G) is defined as

$$\left[f{*}_{G}\psi \right]\left(g\right)=\sum_{c=1}^{N_{c}}\int_{G}f_{c}\left(\tilde{g}\right)\psi_{c}(g^{-1}\tilde{g})d\tilde{g},$$ (4)

$$=\sum_{c=1}^{N_{c}}\int_{G}f_{c}\left(\tilde{g}\right)L_{g}\left[\psi_{c}\right]\left(\tilde{g}\right)d\tilde{g}.$$ (5)

Unlike Eq. 2, the domain of group convolution [f*_G_ *ψ]*, input *f,* and convolution kernel *ψ* in the new model is grouped. In short, group convolution can be considered as a set of inner products between input signal f and group transformation ψ. An important advantage of group convolution is that it extends equivariant (formula 3) to groups; that is, the group convolution defined on groups satisfies the commutative law

$$L_{\tilde{g}}\left[f{*}_{G}\psi \right]\left(g\right)=\left[L_{\tilde{g}}\left[f\right]{*}_{G}\psi \right]\left(g\right),g,\tilde{g}\in G.$$ (6)

**FIGURE 2:**
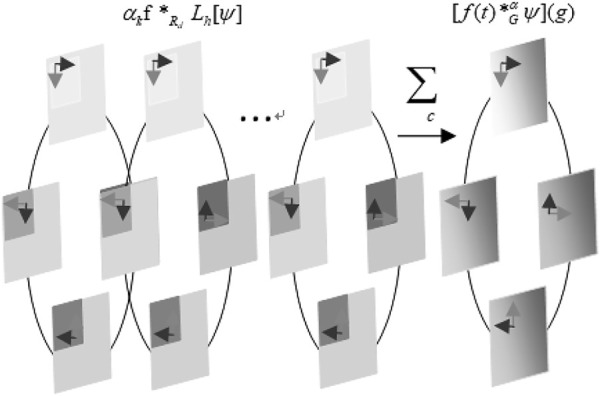
Pruning group convolution on the rotation translation group.

This property is called group equivariant. Like spatial convolution, group convolution is the only linear group equivariant mapping. A rotation translation group (also called the P4 group) is a typical affine group as shown in Figure 2. Group *P4* is defined as $R=\left\{e,r1,r2,r3\right\}$ , where r is 90° rotation. The group convolution is defined as the *|R |*(*|R |* = 4) convolutions *ψ* between the *H*-transform *L*_*r*_ *[ψ]*(*∀r∈R*) of the filter *ψ* and the input *f*. The convolution at each rotation angle in the group convolution is equal to the sum of the spatial channel convolutions $\left[f_{k}\;{*}_{R^{2}}L_{k}\left[\psi_{k}\right]\right]$ between f and $L_{R}\left[\psi \right]$ (group element $\tilde{r}\in R$ and channel $k\in \left[N_{c}\right]$ ).

### 3.2 Dynamic pruning group equivariant maps

In this part, we first introduce the motivation of pruning the group convolution network and then introduce the detailed implementation method.

Group convolution pruning is calculated by pruning operator $\alpha_{k}$ , $\alpha_{k}=h_{k}\left(W_{k}^{\left(i,j\right)}\right)$ . Therefore, pruning group convolution can adjust the connection of group elements *g∈G* on different channels *k∈[N*_*c*_ *]*.

$$\left[f{*}_{G}^{\alpha }\psi ]\left(g\right)=\sum_{k=1}^{N_{c}}\int_{G}\alpha_{k}\left(g,\tilde{g}\right)f_{k}\left(\tilde{g}\right)L_{g}[\psi_{k}]\left(\tilde{g}\right)d\tilde{g}].\right.$$ (7)

#### 3.2.1 Marking

First, we agree on the symbols that appear in this article. It is assumed that the GCNN model can be expressed as *{W*_*k*_ *: 0≤k ≤ C},* where *W*_*k*_ is the connection weight matrix in the *k* ^*th*^ layer. For the fully connected layer with m-dimensional input and n-dimensional output, the size of *w*_*k*_ is m×n。. For the group convolution layer with a trainable kernel, we expand the weight of each convolution kernel into a vector and splice them into a matrix as a whole.

In order to represent the pruning of group convolution networks, we introduce the set $W_{k},T_{k}:0\leq k\leq C$ . Each *T*_*k*_ is a binary matrix, in which each element represents the activation state of the network connection, that is, whether the network connection is deleted. Matrix *T*_*k*_ can be understood as the mask matrix of network weight matrix *W*_*k*_.

#### 3.2.2 Dynamic pruning

The purpose of pruning is to simplify the network and improve the generalization ability of the network. Here, the key to pruning is to delete redundant connections and retain those connections that are critical to network performance. However, in a specific network, the importance of parameters (i.e., the importance of connections) is dynamic due to the interaction and activation of neurons connected to each other. In other words, some seemingly redundant connections will become crucial because of the deletion of other connections around them. Therefore, it is very important to keep the repair ability of the network structure in the process of model training.

Taking the kth layer as an example, dynamic pruning can be transformed into the following optimization problem:

$$\min_{W_{k}{,T}_{k}}\;{L(W}_{k}*T_{k})\;s.t.\;T_{k}^{\left(i,j\right)}=d_{k}\left(W_{k}^{\left(i,j\right)}\right),\forall \left(i,j\right)\in l,$$ (8)

where *L(•)* is the loss function, * is the Dot product operator, the set l is composed of all weights in the matrix *W*_*k*_, and *d*_*k*_ *(•)* is the discriminant function. If the parameter w seems to be the key in the current layer, then *d*_*k*_ *(w)* = 1; otherwise, it is 0. The function d_*k*_ *(•)* is designed empirically. In order to make the description clearer, we introduce the *d*_*k*_ *(•)* function in Section 3.2.3. The problem (8) can be solved by using the random gradient descent (SGD) method to update *w*_*k*_ and *T*_*K*_ alternately, which is described as follows.

In the chain rule of calculating the gradient by using the back-propagation algorithm, the following can be obtained:

$$\nabla_{W^{\left(l\right)}}L=\sum_{i=1}^{n_{l}}\sum_{j=1}^{n_{l-1}}\frac{\partial L}{\partial {\overset{∼∼}{W}}_{ij}^{\left(l\right)}}\cdot \nabla_{W}{\overset{∼∼}{W}}_{ij}^{\left(l\right)}.$$ (9)

Here, ${\overset{∼∼}{W}}_{ij}^{\left(l\right)}=W_{ij}\cdot T$ , ${\overset{∼∼}{W}}_{ij}^{\left(l\right)}$ is only affected by $W_{ij}$ , and

$$\frac{\partial {\overset{∼∼}{W}}_{ij}^{\left(l\right)}}{\partial W_{ij}}=\frac{\partial W_{ij}\cdot T_{ij}}{\partial W_{ij}}=T_{ij}+W_{ij}\cdot \frac{\partial \cdot T_{ij}}{\partial W_{ij}}.$$ (10)

We consider

$$\begin{array}{l} \frac{\partial \log\frac{\left|W_{ij}\right|}{mu}}{\partial W_{ij}}=\left\{\begin{array}{l} \frac{\partial \log\left(-\frac{W_{ij}}{mu}\right)}{\partial W_{ij}},if\;W_{ij}<0\\ \frac{\partial \log\frac{W_{ij}}{mu}}{\partial W_{ij}},if\;W_{ij}\geq =0, \end{array}\right.\\ =\left\{\begin{array}{l} -\frac{1}{W_{ij}},if\;W_{ij}<0\\ \frac{1}{W_{ij}},if\;W_{ij}\geq =0 \end{array}\right.=\frac{1}{W_{ij}}. \end{array}$$ (11)

So, when $W_{ij}$ is pruned (i.e., $\left|W_{ij}\right|\leq \mu$ ),

$$\frac{\partial {\overset{∼∼}{W}}_{ij}^{\left(l\right)}}{\partial W_{ij}}=\left\{\begin{array}{l} \log\varepsilon ,if\;\left|W_{ij}\right|\leq \varepsilon \cdot \mu \\ \log\frac{W_{ij}}{\mu }+\frac{W_{ij}}{\left|W_{ij}\right|},if\;\left|W_{ij}\right|>\varepsilon \cdot \mu . \end{array}\right.$$ (12)

Therefore, the gradient of $W_{ij}$ after dynamic pruning is

$$\begin{array}{l} {\left(\nabla_{W^{\left(l\right)}}L\right)}_{ij}=\frac{\partial L}{\partial {\overset{∼∼}{W}}_{ij}^{\left(l\right)}}\cdot \frac{\partial {\overset{∼∼}{W}}_{ij}^{\left(l\right)}}{W_{ij}},\\ =\{\begin{array}{l} \log\varepsilon \cdot \frac{\partial L}{\partial {\overset{∼∼}{W}}_{ij}^{\left(l\right)}},if\;\left|W_{ij}\right|\leq \varepsilon \cdot \mu \\ \left(\log\frac{W_{ij}}{\mu }+\frac{W_{ij}}{\left|W_{ij}\right|}\right)\cdot \frac{\partial L}{\partial {\overset{∼∼}{W}}_{ij}^{\left(l\right)}},if\;\left|W_{ij}\right|>\varepsilon \cdot \mu \end{array}. \end{array}$$ (13)

When the absolute value of $W_{ij}$ is small relative to μ ( $W_{\text{ij}}\leq \varepsilon \cdot \mu$ ) and ε takes the appropriate value, ${\left(\nabla_{W^{\left(l\right)}}L\right)}_{ij}=\log\varepsilon \cdot \frac{\partial L}{\partial {\overset{∼∼}{W}}_{ij}^{\left(l\right)}}$ . Although the corresponding gradient does weaken a lot, it does not completely disappear and will be updated all the time during the training process. After several iterations, W_ij_ may return to a more moderate size relative to mu so that the pruned connection can be restored.

When the absolute value of the pruned W_ij_ is equal to mu, ${\left(\nabla_{W^{\left(l\right)}}L\right)}_{ij}\approx \frac{W_{ij}}{\left|W_{ij}\right|}\cdot \frac{\partial L}{\partial {\overset{∼∼}{W}}_{ij}^{\left(l\right)}}$ , it will be a very small number so that W_ij_ will not be updated in the gradient descent algorithm. In this case, because W_ij_ itself cannot be changed, the W_ij_ connection may be restored only when the mu itself becomes smaller.

The binary matrix *T*_*k*_ can be solved by formula (12). The traditional gradient descent method can be used to optimize w_k_, and its update is determined by the following formula:

$$\begin{array}{l} W_{k}^{\left(i,j\right)}\;\leftarrow W_{k}^{\left(i,j\right)}-\beta \frac{\partial }{\partial W_{k}^{\left(i,j\right)}T_{k}^{\left(i,j\right)}}\\ L\left(W_{k}⊗T_{k}\right),\forall \left(i,j\right)\in I, \end{array}$$ (14)

where *β* is the learning rate. Here, not only are the non-zero parameters of *T*_*k*_ updated (these updated parameters are considered to be unimportant and ineffective for reducing network loss) but also the zero parameters of T_k_ are updated. This strategy can recover the incorrect pruning.

The partial derivative in formula (14) can be calculated by the chain derivation rule. After repeated iterations, the pruning group convolution network will converge to a higher accuracy, and the generalization ability will be improved. The aforementioned process is shown in Algorithm 1.

Algorithm 1. Group convolution network pruning: gradient descent algorithm in group convolution network pruning.

**Input:** X: training datum; {*W*_*k*_ *: 0 ≤ k ≤ C}*: the reference model; α: base learning rate; *μ*: mean value; σ: standard deviation; *γ*: the multiple of the threshold value of the pruned weight compared with the standard deviation *σ;* ε: the minimum value to ensure the stability of weight; f: learning policy.
**Output:** {*W*_*k*_ *, T*_*k*_ *: 0 ≤ k ≤ C*}: the updated parameter matrices and their binary masks.
 { *W*_*k*_ * T_*k*_ *: 0 ≤ k ≤ C*}: Hadamard product of the updated parameter matrix and its binary masks.
  *W*_*k*_ *← W*_*k*_, *T*_*k*_ *← 1* is initialized ∀*0 ≤ k ≤ C*, β ← 1, and iter ← 0
Repeat
 Small-batch samples are randomly selected as input X.
 The value of the loss function is calculated with (*W*_*K*_⊙*T*_*k*_).
 Backward propagation of the model output and the gradient of the loss function is calculated by Eq. 9.
 While (*k* < *C*),
   T_k_ is updated by Eq. 6 and the current W_k_.
   *W*_*k*_ is updated by Eq. 14 and the current loss function gradient by Eq. 9
 Iter ← iter+1 is updated until the iter reaches its desired maximum.

**FIGURE 3:**
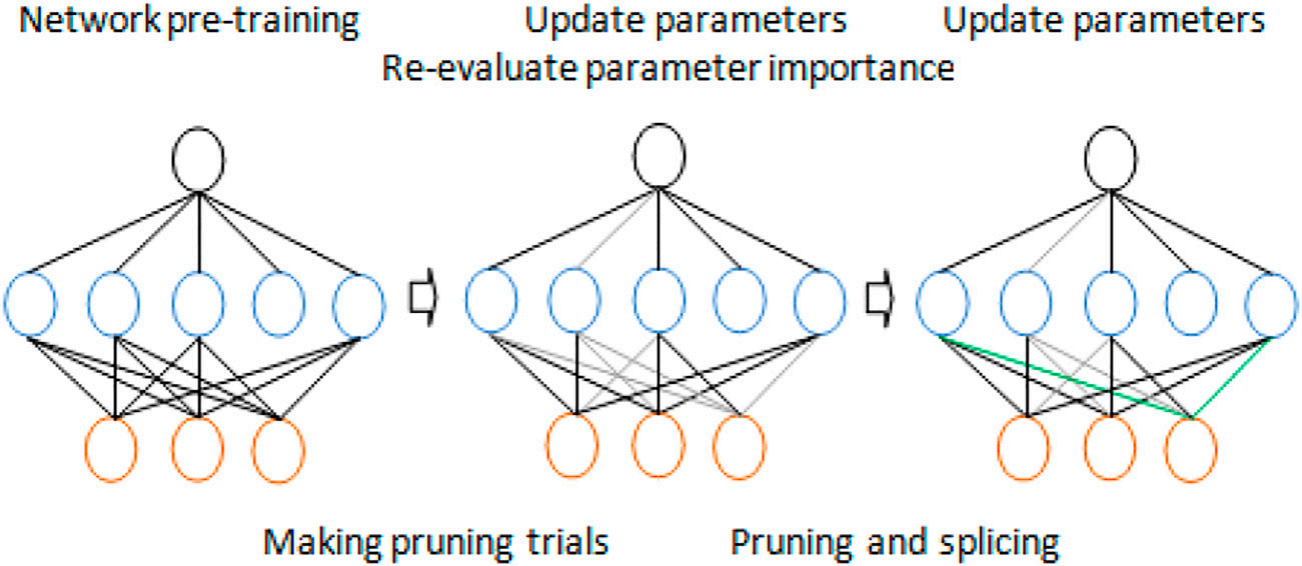
Dynamic network pruning for models with parameter redundancy.

Dynamic pruning has two meanings. First, in the current state, redundant connections will be deleted through iteration. However, on the other hand, if incorrectly trimmed connections once seemed important, they should be reestablished. An overview of our approach is shown in Figure 3.

#### 3.2.3 Parameter weight

Since the measurement of parameter importance affects the state of network connection, the functions d_k_(•),∀0 ≤ k ≤ C are essential for our dynamic network pruning. We tested several candidates and found that the absolute value of the weight is the best option.

One of the core issues of pruning is to determine which weights are crucial to the network and need to be retained and which weights are redundant and need to be deleted. Through experiments, we find that the absolute value of weight can be used as a measure of connection importance. In each iteration of Algorithm 1, the parameters with relatively small amplitude are pruned temporarily, and the parameters with larger amplitude are retained or spliced. Obviously, the threshold has a great influence on the compression ratio of the group convolution neural network. For each layer of the group convolution network, the corresponding threshold is set according to the average absolute value and variance of its connection weight.

$$\begin{array}{l} d_{k}\left(W_{k}^{i,j}\right)=\log\left(\max|\left(\varepsilon ,\frac{\left|W_{k}^{i,j}-\mu \right|}{\gamma \cdot \sigma }\right)|,\right.\\ \mu =\frac{1}{m*n}\sum_{i=1}^{m}\sum_{j=1}^{n}\left|W^{i,j}\right|,\\ \sigma =\sqrt{\frac{1}{m*n-1}\sum_{i=1}^{m}\sum_{j=1}^{n}{\left(\left|W^{i,j}\right|-\mu \right)}^{2}.} \end{array}$$ (15)

*μ* and σ represent the mean value and standard deviation of the absolute value of each element in the weight matrix W_k_, respectively. *γ* is the multiple of the threshold value of the pruned weight compared with the standard deviation *σ* of the absolute value of each element in the weight matrix, and ε is the minimum value to ensure the stability of the weight.

## 4 Experimental results and analysis

### 4.1 Emotiv EEG dataset classification experiment

#### 4.1.1 EEG data acquisition experiment

**FIGURE 4:**
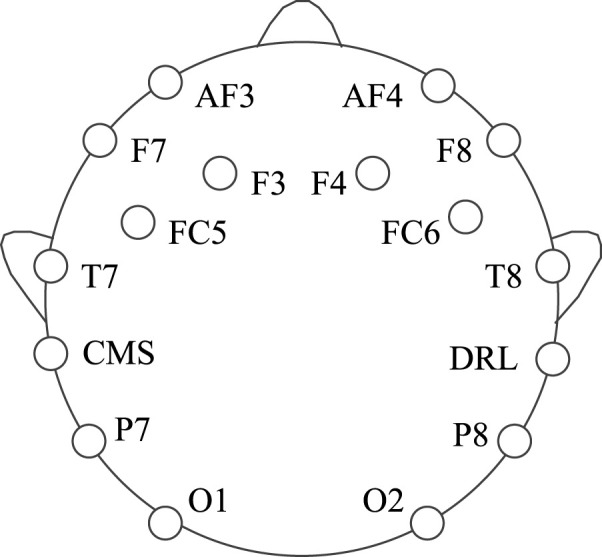
Electrode position of the EmotivPRO EEG acquisition instrument.

In this experiment, dataset 1 is collected using the Emotiv EEG acquisition instrument developed by Emotiv Systems in the United States (the offline experiment of a lower computer such as a wheelchair is carried out using Emotiv EEG acquisition instrument). Its main components include an electrode cap, electrode, electrode box, and Emotiv wireless USB receiver. The electrode cap contains an amplifier, ADC, filter (0.2–45 Hz), and notch filter (except power frequency interference). One electrode cap has 16 electrodes, two of which are reference electrodes, and the rest are for acquisition, as shown in Figure 4. At the same time, we set the electrode according to the international 10–20 standard electrode placement method, and the sampling frequency is 128 Hz.

**FIGURE 5:**
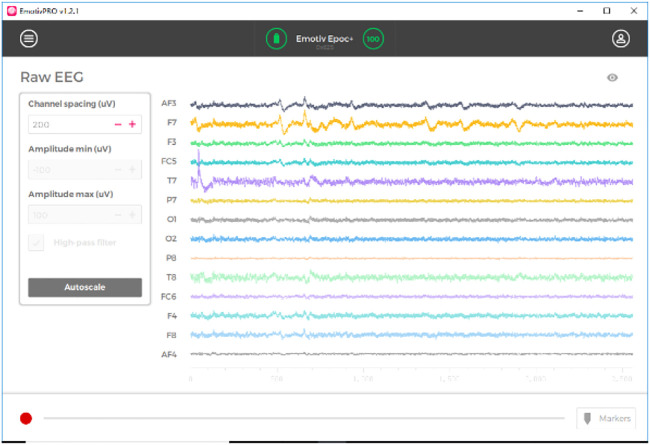
EmotivPRO interface.

The EmotivPRO sensor collects, displays, and saves the EEG signals of each channel through its own software application emotivPRO (acquisition interface is shown in Figure 5).

In this paper, we will study the EEG signals of left and right hand motor imagery. The specific steps of collecting EEG signals are as follows: first, the subjects are calmed down and kept relaxed for 40 s, and then, the acquisition process is started. The subjects are relaxed for 6 s; then, there will be a prompt tone in 6 s to prompt the subjects to start imagining left or right hand movement, and there will be a prompt tone in 10 s to prompt the subjects to stop imagining. Taking 1 s as a group of EEG sample data, four groups of EEG sample data can be obtained by collecting once. Then, the aforementioned acquisition process is repeated until there are 180 groups of left hand and right hand motor imagery EEG signals; that is, there are a total of 360 groups of EEG signal sample data.

In order to reduce the amount of data, this paper only selects the F3, F4, FC5, FC6, T7, and T8 channel signals located in the motor sensory area for analysis and divides the datasets into training sets and test sets according to the ratio of 4:1.

#### 4.1.2 Data preprocessing process

The signal-to-noise ratio of the collected EEG signal is very low, which usually contains significant background noise, such as clutter, electrooculogram, ECG, and EMG. In this paper, the EEG signal is preprocessed as follows.

Step 1 Removing the abnormal samples: Due to environmental noise, poor equipment contacts, and other factors, some incorrect samples will be produced when the EEG signals are collected. Therefore, the average potential of the channel is used as the reference value for comparison with each sample data, which eliminates the large difference.

Step 2 Normalizing to average: By subtracting the amplitude of each sample from its average amplitude, the average value of the EEG signal can be 0, and the analysis process is easier.

Step 3 Band-pass filtering: When imagining the movement of the left or right hand, event-related synchronization/desynchronization is mainly manifested as μ rhythm (6–13 hz) and β rhythm (14–30 hz), so the EEG signal is filtered by a 6–30 hz band-pass filter.

Step 4 Short-time Fourier transform.

The 2008 BCI competition IV dataset 2B is taken as an example, which includes the records of three electrodes (C3, CZ, and C4) in the left/right hand MI task. These electrodes are located in the motor area of the brain.

Pfurtscheller and Da Silva (1999) showed that the Mu band (6–13 Hz) energy observed in the motor cortex decreased by performing MI tasks. This reduction is called event-related desynchronization (ERD) (Yu et al., 2015). MI tasks also result in increased energy in the β-band (14–30 Hz), which is called event-related synchronization (ERS). Left-handed and right-handed motor MI tasks caused ERD and ERS in the left and right sides of the motor cortex, respectively, which affected the EEG signal intensity of C3 and C4 electrodes. CZ was also affected by the hand movement MI task. Considering these facts, we design network input to take advantage of the time and frequency characteristics of the data.

STFT is applied to a time series with a duration of 2 s. The window size of the STFT is 64 and the time interval is 14. From sample 1 to sample 1,000, the STFT is calculated for 1,000 samples of 67 windows. Then, mu and beta bands are extracted from the output spectrum. The band between 6–13 and 17–30 is considered to represent mu and beta bands, respectively. The size of the extracted image in the mu band is 16 × 67, and that of the Beta band is 15 × 67. These images are then combined into an N_fr_ × N_t_ image, where N_fr_ = 31 and N_t_ = 67.

**FIGURE 6:**
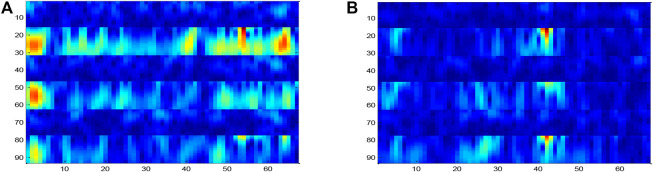
Input sample image, including two frequency bands of C3, CZ, and C4 for each electrode.

The process was repeated for three electrodes (C4, CZ, and C3). The results were combined in the way of preserving the information of electrode adjacency. The resultant size of the input image was N_h_ × N_t_, where N_h_ = N_c_ * N_fr_ = 93. A sample input signal time–frequency diagram constructed for the right hand MI task experiment is shown in Figure 6. By using this method, the left and right sides of the motor cortex are activated, and different activation patterns are generated along the vertical cortex.

The ERD effect in channel C3 is clearly shown in Figure 6A, which corresponds to the right MI task (the 6–13 Hz band in channel C3 is darker than that in channel C4). However, the ERS effect is not obvious on the C3 electrode. Similar to the right hand, for samples collected for the left hand MI task, this activation is expected to occur on the opposite side of the electrode as shown in Figure 6B. The time–frequency diagram is constructed for each test sample and used as the input of the DPGEN in the next stage.

#### 4.1.3 Determination of threshold

In the DPGEN algorithm, *γ* is the multiple of the threshold value of the weight pruned compared with the standard deviation *σ* of the absolute value of each element in the weight matrix.

The EEG data of the first person are taken as the experiment, and the value is evenly taken at the interval of 1 between 1 and 10. The processed samples are input into the DPGEN. The results show that when the value of *γ* exceeds 8, the recognition accuracy of the EEG signal decreases rapidly.

**FIGURE 7:**
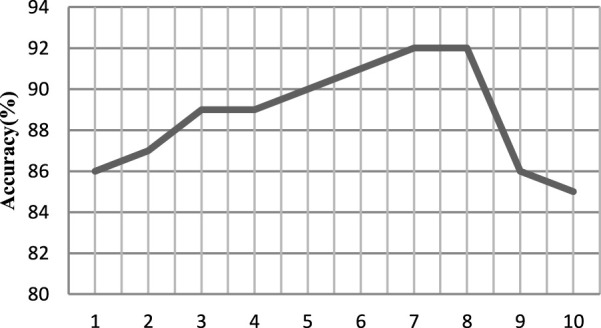
Recognition accuracy under a different threshold γ.

As shown in Figure 7, when the threshold γ is 7 and 8, the accuracy is relatively high, so the size set in this paper is 8.

#### 4.1.4 Experimental results and analysis

**FIGURE 8:**
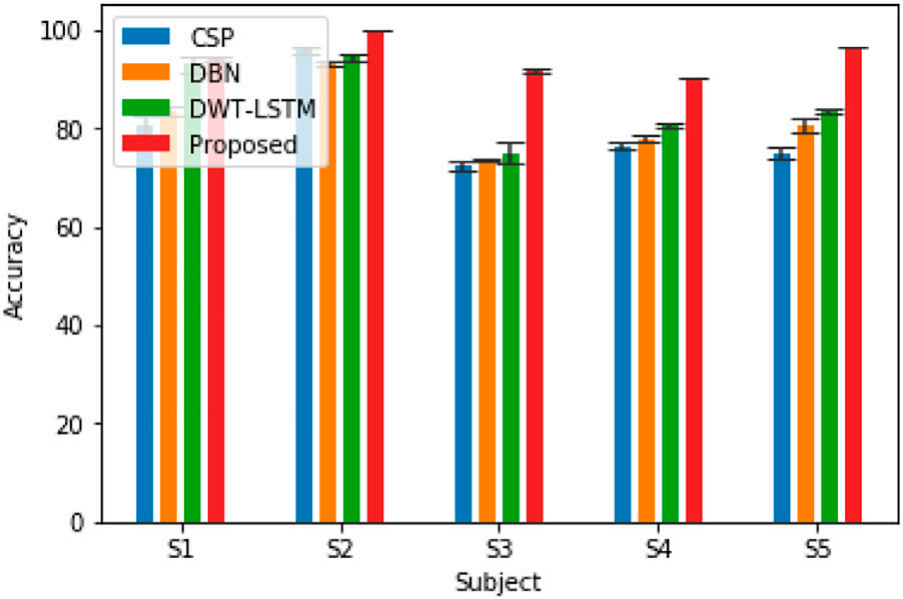
Accuracy of EEG recognition algorithms for five different subjects.

In order to verify the effectiveness of the pruning group convolutional neural network (DPGEN) proposed in this paper, this study conducts a comparative experiment with the left and right hand motor imagery EEG dataset collected by Emotiv and compares its recognition accuracy with the classical EEG feature extraction algorithms CSP (Wu et al., 2013), DBN (Chu et al., 2018), and DWT-LSTM (Li et al., 2016). In the comparative experiment, the CSP used the eigenvectors corresponding to the first two eigenvalues as the spatial filter and used the RBF kernel SVM as the classifier. The results of accuracy are shown in Figure 8.

As shown in Figure 8, compared with the results of the CNN, STFT-CNN, and DPGEN, the recognition accuracy of the DPGEN is higher, which indicates that the DPGEN can make full use of the effective information in EEG signals. The recognition accuracy of the DPGEN method in this paper is higher than that of the other methods in the data of five subjects, and the variance of prediction accuracy of this method is smaller than other methods’. It shows that the proposed method has better stability.

We need to verify whether the performance improvement of the proposed method is statistically significant compared with the other three algorithms. In this experiment, two-way analysis of variance (ANOVA2) test was used to calculate the *p*-value between the proposed methods and these methods. Subjects and methods are two independent variables of the test, and classification accuracy is the dependent variable of the test. The least significant difference (LSD) method was used for multiple comparisons. Table 1 lists the *p*-values between the proposed algorithm and the other three algorithms. It is generally considered that when the *p*-value is less than 0.05, and there is a significant difference between the performances of the two algorithms involved in the comparison. As shown in Table 1, the *p*-value between the proposed algorithm and CSP, DBN, and DWT-LSTM is less than 0.05. Therefore, the improvement in the recognition accuracy of the algorithm proposed in this paper is significant.

**TABLE 1:** P-values between the proposed method and other three algorithms.

Method	Statistic	CSP	DBN	DWT-LSTM
*P*-values	Mean	<0.008	<0.002	<0.003
*P*-values	Variance	<0.004	<0.028	<0.034

In order to compare the performance of the pruning group convolution network using different group convolution kernels, we conducted further experiments on the dataset collected by the laboratory. First, a CNN architecture with pruning is constructed, including two layers of 3 × 3 convolution, 24 channels in each layer, relu activation function, batch normalization, and dropout.

Next, we replace each convolution of the pruned convolutional neural network (PCNN) with p4 convolution (Eqs 10, 11), and the generated feature maps are composed of features that change in rotation. The number of filters is set to 6*4 = 24 to keep the number of parameters roughly fixed compared to the CNN (the number of channels is 24). The accuracy of the P4CNN is somewhat improved compared to the standard CNN with pruning.

Then, we tested the pruning group convolutional network proposed in this article, replacing each convolution of the pruning convolutional network with a P4M group convolution kernel (Eqs 10, 11), and the generated feature map is changed by rotation and mirroring. It has characteristic composition and the same transformation law as the input signal. The performance of this network is better than that of the CNN with pruning and PP4GCN. The reason may be that P4M group convolution adds mirroring and rotation transformations in the middle layer.

**FIGURE 9:**
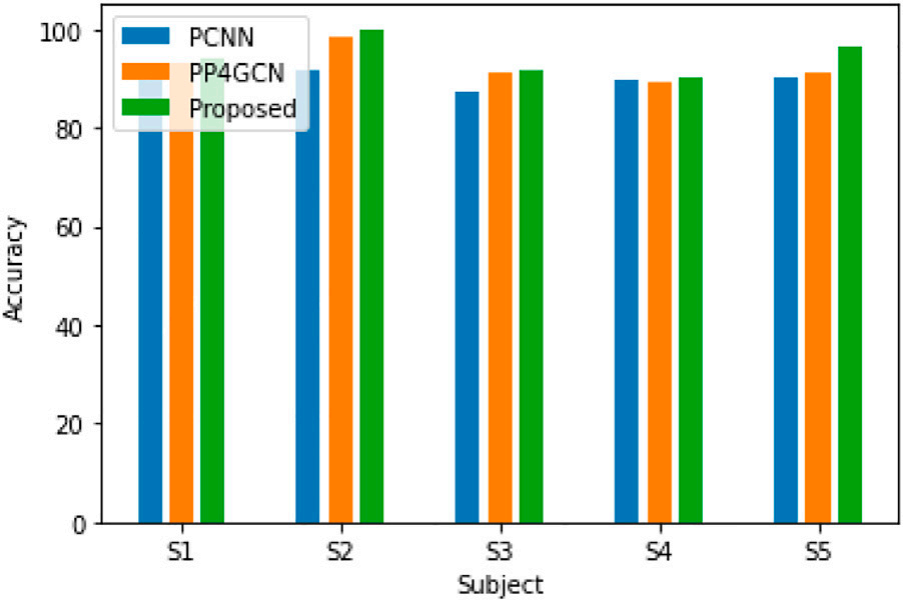
Recognition accuracy for five different subjects (with different group convolution kernels).

In another experiment, we use the proposed DPGEN and GCNN to classify motor imagery EEG signals. The input EEG signal is also preprocessed by short-time Fourier algorithm. DPGEN performed well in all five subjects, indicating that the hierarchical features extracted from the extracted EEG signals through different group convolution kernels contain abundant information related to classification tasks, which can improve the recognition accuracy of EEG signals, as shown in Figure 9.

**FIGURE 10:**
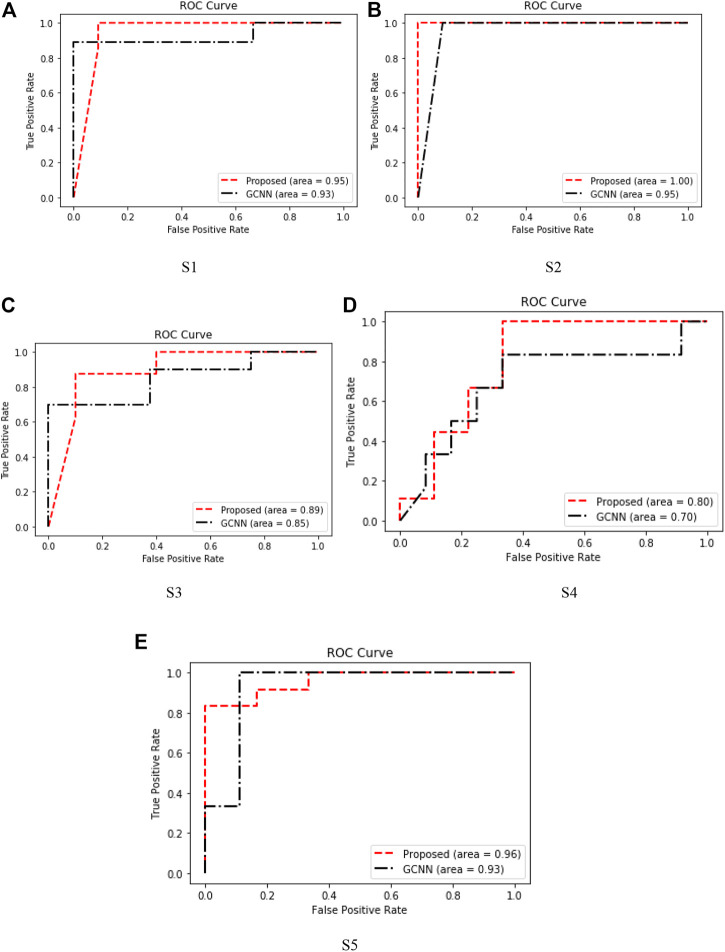
ROC curves of five subjects. **(A)** S1. **(B)** S2. **(C)** S3 **(D)** S4. **(E)** S5.

The receiver operating characteristic (ROC) curve in the figure is a graphical representation used to evaluate the performance of the classification model. The performance of the algorithm can be judged by analyzing the value of the area under the curve (AUC). As shown in Figure 10, in most cases, the method proposed in this paper obtains higher AUC value than GCNN, which shows that our method has more advantages in EEG motor imagination signal processing.

The DPGEN performed well in all five subjects, indicating that the hierarchical features extracted from the extracted EEG signals through different group convolution kernels contain abundant information related to classification tasks, which can improve the recognition accuracy of EEG signals as shown in Figure 9.

At the same time, we will analyze the model compression performance of the proposed method. In order to compare fairly and copy conveniently, we follow the default experimental settings of the SGD method, including training batch size, basic learning rate, learning strategy, and maximum training iterations. A brief summary of the compression results is shown in Table 2.

**TABLE 2:** Dynamic network pruning can significantly reduce the model complexity of the GCNN, while the prediction error rate can be reduced to a certain extent.

Model	Average accuracy	Parameters (K)	Iterations (K)	Compression
GCNN	81.33	113	10	
DPGEN	83.93	7.5	12	15×

### 4.2 Public dataset BCI competition IV 2B recognition experiment

This paper uses the open dataset BCI competition IV 2b of the fourth BCI competition for further verification. The dataset contains nine subjects’ left and right hand motor imagery EEG data, each subject collected five times, the first two collected each time contains 120 groups of data, and there is no feedback, while the last three collected each time contains 160 groups of data, and there is feedback; that is, each subject collected a total of 720 groups of experimental data. In the process of acquisition, EEG signals of C3, CZ, and C4 channels were recorded, and the sampling frequency was 250 Hz. The collected signals were filtered by a 0.5–100-Hz bandpass filter and 50 Hz notch filter.

In this part, in order to adapt to the format changes of the EEG data, the dataset is preprocessed similar to the previous dataset. Some parameters of the DPGEN are set as follows: group convolution kernel 1:3 × 3, group convolution kernel 2: 3 × 3, pooling layer 1:2 × 1, and pooling layer 2:2 × 1.

**FIGURE 11:**
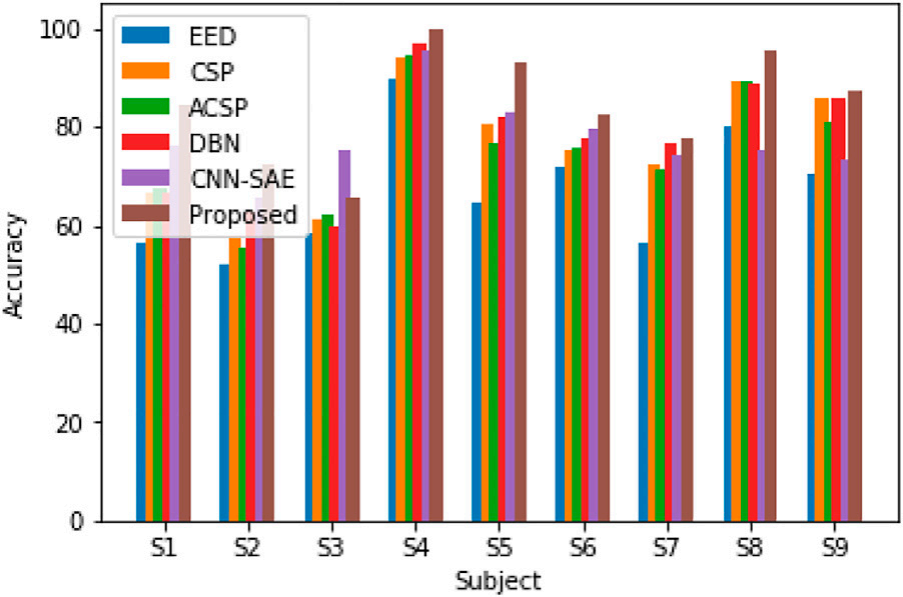
Recognition accuracy of different algorithms on the BCI competition IV 2B dataset.

In order to verify the recognition effect of the DPGEN EEG recognition method on the open dataset BCI competition IV 2B, the recognition accuracy is compared with the top three results of BCI competition EED (Sun, 2010), CSP, ACSP (Sun and Zhou, 2014), DBN, and CNN-SAE (Tabar and Halici, 2016). The comparison results are shown in Figure 11.

Figure 11 shows that the DPGEN method proposed in this paper has better results in the data of most subjects in the open dataset BCI competition IV 2B. Due to the individual differences of the EEG signals, the recognition accuracy of data from very few subjects is not the highest. However, compared with other methods, the average recognition accuracy of this method is the highest, which shows the effectiveness of this method.

The *p*-values between the proposed algorithm and other eight algorithms are shown in Table 3. It can be seen that the *p*-values between the proposed algorithm and EED, CSP, ASCP, DBN, and CNN-SAE are less than 0.05.

**TABLE 3:** P-values between the proposed method and other five algorithms.

Method	EED	CSP	ACSP	DBN	CNN-SAE
*P*-value	<0.001	0.003	<0.002	0.008	0.042

### 4.3 Online experiment

**FIGURE 12:**
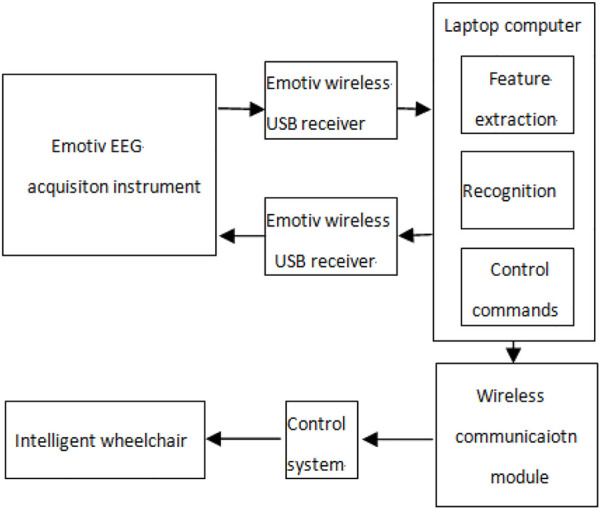
Structure of the intelligent wheelchair system.

In order to verify the performance of the algorithm in EEG real-time processing, we conducted an online test on the self-designed intelligent wheelchair system. The system mainly includes the following subsystems: the Emotiv EEG acquisition instrument, portable computer, wireless communication module, control system, and wheelchair. The structure of the intelligent wheelchair system is shown in Figure 12.

The Emotiv EEG acquisition instrument shows good performance in the acquisition of EMG and EEG signals. Through previous experiments, we found that the muscle action of “gritting teeth” can produce obvious voltage changes in the F8 channel, so this signal is used to start and stop online experiments. The action of “blinking” will produce an obvious voltage change in the FC4 channel, which can be used to generate the wheelchair straight command. In addition, the motor imaginary EEG signals of six channels F3, F4, FC5, FC6, T7, and T8 are collected. The left hand and right hand motor imaginary EEG signals are classified by the method proposed in this paper, which are used to generate the commands of the wheelchair turning left and right, respectively.

The first three subjects in our laboratory’s self-collected dataset were used to carry out the online experiment. The commands that the wheelchair needs to perform include left turn, right turn, and straight ahead. All kinds of experiments are carried out in a cross-way. Each type of experiment was conducted 140 times, and the rest was 5 min after every 20 experiments, with an interval of 20 s. The recognition accuracy of the wheelchair online control experiment is shown in Table 4.

**TABLE 4:** Online recognition accuracy (%) of three subjects.

Subject	Straight	GCNN			Proposed		
		Left	Right	Average	Left	Right	Average
S1	93.26	78.79	76.53	77.66	82.46	82.18	82.32
S2	87.54	88.43	86.57	87.5	92.10	90.89	91.50
S3	95.56	83.37	85.34	84.36	88.89	87.46	88.18

Table 4 shows that the recognition accuracy of this method is higher than that of the GCNN model. Because the EMG signal has more obvious characteristics than the EEG signal, the online recognition accuracy of the EMG signal is higher than that of the EEG signal. In addition, by comparing the experimental results given in Figure 6 and Table 4, it can be found that in general, the online recognition accuracy is lower than the offline recognition accuracy. This is because online experiments may be subject to more factors. For example, subjects may be vulnerable to the influence of the surrounding environment and fatigue.

## 5 Conclusion

In this paper, we proposed a framework for motor imagery EEG recognition based on the dynamic pruning group equivariant network. The proposed framework can integrate the intrinsic relationship of EEG signals of various EEG channels. Combined with multilevel features extracted from different group convolution layers, dynamic pruning reduces the number of parameters, reduces the complexity of the network, and improves the recognition accuracy of small sample data. Finally, experiments are carried out on the BCI IV 2B dataset and laboratory self-collected dataset. In the future, we will use domain adaptation and domain generalization to study the effectiveness of topic-independent EEG-based motor imagery EEG recognition in the group equivariant framework, integrate structure and regularity into the process of weight pruning, and establish a unified framework of weight pruning, activation reduction, and weight clustering.

## Data Availability

The original contributions presented in the study are publicly available. This data can be found here: Publicly available datasets were analyzed in this study. These data can be found at: http://bbci.de/competition/iv/download.
